# Energy, ageing, fidelity and sex: oocyte mitochondrial DNA as a protected genetic template

**DOI:** 10.1098/rstb.2012.0263

**Published:** 2013-07-19

**Authors:** Wilson B. M. de Paula, Cathy H. Lucas, Ahmed-Noor A. Agip, Gema Vizcay-Barrena, John F. Allen

**Affiliations:** 1 School of Biological and Chemical Sciences, Queen Mary University of London, Mile End Road, London E1 4NS, UK; 2 Ocean and Earth Science, National Oceanography Centre Southampton, University of Southampton, European Way, Southampton SO14 3ZH, UK; 3 Centre for Ultrastructural Imaging, King's College London, New Hunt's House Guy's Campus, London SE1 1UL, UK; 4 Research Department of Genetics, Evolution and Environment, University College London, Gower Street, London WC1E 6BT, UK

**Keywords:** cytoplasmic inheritance, maternal inheritance, *Aurelia aurita*, mitochondrial genome, oxidative phosphorylation, aging, Weismann barrier

## Abstract

Oxidative phosphorylation couples ATP synthesis to respiratory electron transport. In eukaryotes, this coupling occurs in mitochondria, which carry DNA. Respiratory electron transport in the presence of molecular oxygen generates free radicals, reactive oxygen species (ROS), which are mutagenic. In animals, mutational damage to mitochondrial DNA therefore accumulates within the lifespan of the individual. Fertilization generally requires motility of one gamete, and motility requires ATP. It has been proposed that oxidative phosphorylation is nevertheless absent in the special case of quiescent, template mitochondria, that these remain sequestered in oocytes and female germ lines and that oocyte mitochondrial DNA is thus protected from damage, but evidence to support that view has hitherto been lacking. Here we show that female gametes of *Aurelia aurita*, the common jellyfish, do not transcribe mitochondrial DNA, lack electron transport, and produce no free radicals. In contrast, male gametes actively transcribe mitochondrial genes for respiratory chain components and produce ROS. Electron microscopy shows that this functional division of labour between sperm and egg is accompanied by contrasting mitochondrial morphology. We suggest that mitochondrial anisogamy underlies division of any animal species into two sexes with complementary roles in sexual reproduction. We predict that quiescent oocyte mitochondria contain DNA as an unexpressed template that avoids mutational accumulation by being transmitted through the female germ line. The active descendants of oocyte mitochondria perform oxidative phosphorylation in somatic cells and in male gametes of each new generation, and the mutations that they accumulated are not inherited. We propose that the avoidance of ROS-dependent mutation is the evolutionary pressure underlying maternal mitochondrial inheritance and the developmental origin of the female germ line.

## 1. Mitochondria and mitochondrial DNA

Mitochondria are the double-membrane-bounded bioenergetic organelles found in the cytoplasm of most eukaryotic cells. Their outer membrane allows free passage of different solutes by a variety of intrinsic carriers. Their inner membrane, in contrast, is a broadly impermeable insulating layer that harbours the respiratory chain and separates the inter-membrane space (the electrochemically positive or P-phase) from the mitochondrial matrix (the electrochemically negative or N-phase) during chemiosmotic ATP synthesis [1]. The mitochondrial matrix is homologous to the bacterial cytoplasm, harbouring a genome, 70S ribosomes and a complete apparatus of gene expression [2,3] that amply document the proteobacterial origin of the organelle via endosymbiosis [4–7]. Mitochondrial DNA, first observed in chick embryo [8] and fungi [3,9] and sequenced for humans [10], is typically small. In metazoa, mitochondria contain 13 protein-coding genes for subunits of the energy-transducing electron transport chain of the mitochondrial inner membrane [11]. The overwhelming majority of mitochondrial proteins are nuclear encoded, synthesized as precursors on cytosolic ribosomes and imported into the organelle [12,13]. This paper deals with the biological reasons behind the retention of a few protein-coding genes in mitochondria and the evolutionary consequences thereof for animal development and ageing.

## 2. Cytoplasmic, maternal, non-Mendelian inheritance: why are there genes in mitochondria?

**Figure 1.**
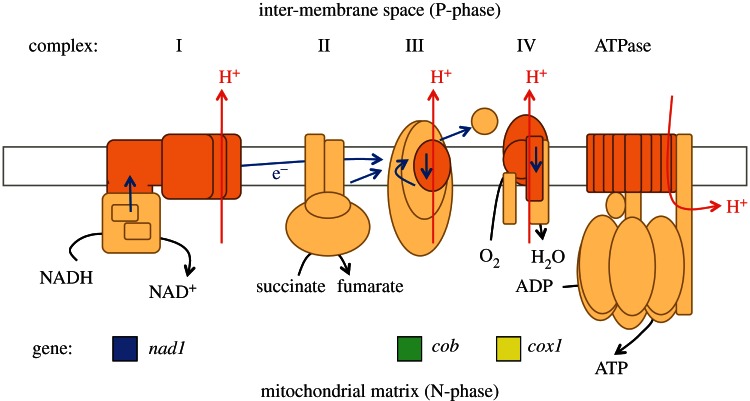
Outline diagram of a mitochondrial inner membrane. Respiratory chain protein complexes I, II, III and IV and the coupling ATPase are plugged through the membrane, which is represented by a horizontal rectangle. In transfer of electrons (e^–^), complexes I, III and IV transport protons (H^+^) outwards, across the membrane, from the mitochondrial matrix (N-phase) to the inter-membrane space (P-phase) and so generate a transmembrane proton gradient that provides energy for synthesis of ATP, which is coupled to proton re-entry into the matrix. Polypeptide subunits are depicted schematically and colour coded to indicate that the proton-translocating complexes contain subunits (dark orange) that are encoded in mitochondrial DNA as well as subunits (peach) that are encoded in nuclear DNA. The three mitochondrial genes chosen for this study encode individual subunits of complexes with which their gene names are aligned vertically: *nad1* (complex I), *cob* (complex III) and *cox1* (complex IV). Adapted from [14].

Figure 1 is a schematic outline of an animal mitochondrial inner membrane. Five multi-subunit protein complexes are involved in the energetic coupling of respiratory electron transport to synthesis of ATP—the process of oxidative phosphorylation. Complexes I, III and IV of the electron transport chain, which transports protons across the inner membrane, and the ATPase, which harnesses that proton gradient, are chimeras, with some subunits synthesized on mitochondrial ribosomes and others imported from the cytosol. This apparently untidy arrangement of separate gene locations leads to two different modes of inheritance. Nuclear genes segregate in a Mendelian manner, while genes in mitochondrial DNA (mtDNA) are cytoplasmically inherited in a non-Mendelian manner. In animals, mtDNA is usually transmitted uniparentally through the maternal line [2,15–17].

Chloroplasts of plants also carry DNA, and are agents of cytoplasmic inheritance for the same reason. Like mitochondria, chloroplasts transduce energy and use a proton-motive force—albeit light driven through photosynthesis—as the intermediate between electron transport and ATP synthesis [18]. In mitochondria and chloroplasts, there is a correlation between membrane-intrinsic proton-motive electron transport and retention of a genetic system, derived originally from a prokaryotic endosymbiont ancestor [19]. Indeed, mitochondria that no longer perform oxidative phosphorylation relinquish their DNA [20–23].

Mitochondria and chloroplasts both stem from bacterial ancestors with large genomes; import the majority of their proteins; and the DNA of both organelles is highly reduced in size. However, in a striking case of multiple convergent evolution, the independent evolutionary reduction of two different bioenergetic organelles has arrived at exactly the same endpoint: a few membrane-associated components of the energy-harnessing electron transport chain and core components of the 70S ribosomes permitting the former's synthesis [14]. This convergence has occurred independently, in parallel, in countless eukaryotic lineages. Can any selective pressure account for this massively parallel evolution towards the same gene sets for two independently derived bioenergetic organelles, and across all known eukaryotic lineages? If the ancestral endosymbiont eventually donated the great majority of surviving genes to the nucleus of the host cell, then why do any genes at all remain?

Among the many proposals that have been put forward to account for the retention of genomes and genetic systems in mitochondria and chloroplasts [12,24–29], one hypothesis applies equally to mitochondria and chloroplasts [19]. It posits that the retention of a given gene within a bioenergetic organelle is the result of natural selection, whereby the corresponding selective advantage for the individual organelle is the ability of the organelle to sense and respond to changes in the redox state of its bioenergetic membrane by being able to regulate the synthesis of proteins in the electron transport chain by means of gene expression. This hypothesis, now named ‘CoRR’ for ‘co-location for redox regulation’ [14,24], mechanistically links (a) the main benefit of bioenergetic organelles, namely energy-harvesting redox chemistry, with (b) their main evolutionary risk, namely generation of hazardous ROS if redox chemistry is unregulated, while accounting for the circumstance (c) that (b) can only be avoided if genes involved in the synthesis of functional complexes in the electron transport chain reside in the same cellular compartment as their gene products [14,24].

There is, so far, more evidence for CoRR in chloroplasts [30,31] than in mitochondria [32]. However, the absence of evidence is not evidence of absence, and predictions of CoRR for mitochondria are clear [33]. In particular, the prokaryotic-derived chloroplast redox sensor kinase has now been identified [34]. As further predicted, this chloroplast sensor kinase has an essential role in photosynthetic control of chloroplast DNA transcription [18,34,35]. CoRR predicts that a mitochondrial redox sensor kinase plays a corresponding role in redox control of respiratory electron transport through effects on mitochondrial gene expression.

## 3. The mitochondrial theory of ageing

As outlined above, mitochondria are a potentially disastrous location for any genome, however small, because of the mutational effects of ROS.

Energy-conserving aerobic respiratory electron transport terminates with concerted four-electron reduction of molecular oxygen to give two molecules of water, a reaction usually coupled to proton translocation across the inner membrane and catalysed by the enzyme cytochrome c oxidase. However, molecular oxygen is also readily reduced, at a number of different points in the respiratory chain, by transfer of a single electron, in which case the product is the superoxide anion radical, O_2_^•−^ [36–40]. Superoxide is a short-lived free radical that reacts rapidly with any of a wide range of chemical substrates, including itself. Superoxide engages spontaneously in disproportionation, or dismutation, a reaction that is also catalysed by the enzyme superoxide dismutase [41]. Superoxide dismutase is ubiquitous in aerobic organisms and also present in many anaerobes; in all cases, it is thought to provide a degree of protection from oxygen toxicity [42–45]. The products of the reaction are oxygen and peroxide, O_2_^2–^, which becomes protonated to give hydrogen peroxide, H_2_O_2_, at neutral pH.

These and other ROS chemically modify cellular constituents, including lipids [46], proteins [47] and nucleic acids, the latter including production of thymine dimers [48]. Because of those reactions, the univalent reduction of oxygen is both cytotoxic and mutagenic. Where ROS react with mitochondrial DNA, the result may be a modified respiratory chain protein that becomes more prone to univalent reduction of oxygen, thus increasing the frequency of mutation. In this way, it is proposed that a vicious circle of self-augmenting mutational load in mitochondrial DNA may be a primary cause of ageing and of many of its associated degenerative diseases [49–53], including cancer [54].

It is, however, still debated whether the mitochondrial theory of ageing, as previously proposed, is central to the ageing process, part of a multi-factorial mechanism, or perhaps not related to ageing at all [55,56]. Some studies suggest that ageing-related mtDNA mutations may arise from DNA replication errors rather than ROS-induced damage [57], which may subsequently trigger downstream apoptotic markers rather than ROS production [58]. Other work proposes that ageing processes induced by mtDNA mutations do not involve ROS production [59,60]. Conversely, recent studies support the mitochondrial theory of ageing [61–69].

If the mitochondrial theory of ageing is correct, it then raises a second problem—how is mitochondrial DNA transmitted into each successive generation in a form that is uncorrupted by the mutations that have accumulated in the parents by the time they reach reproductive age? In short, why is ageing not inherited?

## 4. Separate sexes and mitochondrial division of labour

**Figure 2.**
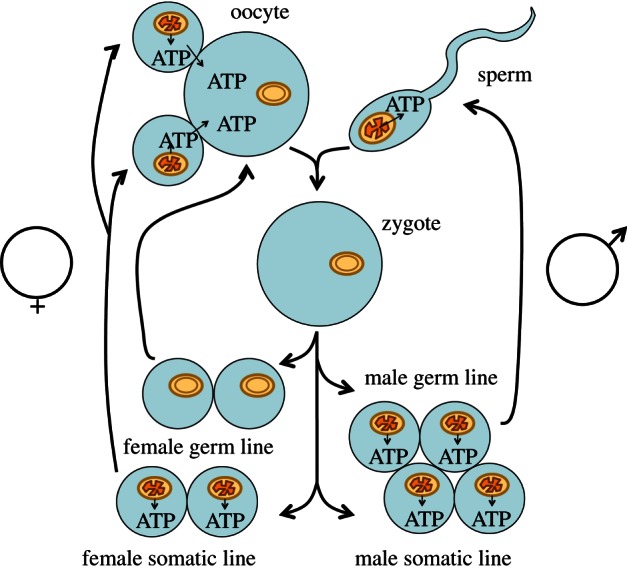
Hypothesis: sequestration of mitochondria of the female germ line as genetic templates, inactive in ATP synthesis. After fertilization, a female gamete (oocyte) carries template mitochondria into the zygote, while the ATP-producing sperm mitochondrion fails to survive, its task completed. During subsequent development, the female germ-line retains only the template mitochondria inherited from the mother. These template mitochondria never differentiate into ATP-synthesizing mitochondria, but perpetuate undamaged copies of mitochondrial DNA. In contrast, the male germ-line and both female and male somatic cell lines develop energetically functional mitochondria, with inner membranes as depicted in figure 1, and which perform ATP synthesis. In contrast, female gametes and female germ cells never synthesize ATP by oxidative phosphorylation, incur no penalty of mutation initiated by respiratory electron transport, and their mitochondria are transmitted from mother to daughter. Adapted from [70].

As a solution to this problem, it has been proposed that oocytes contain genetically repressed, template mitochondria that are retained within the female germ line to be transmitted, in the oocyte cytoplasm, between generations [70]. According to this hypothesis, sperm mitochondria are subject to ROS-induced mutagenesis since they are required to produce ATP for motility. If sperm mitochondrial DNA were retained, after fertilization, by the zygote, then males would transmit damaged mitochondrial DNA to their offspring. Male gametes and somatic cells of both sexes are predicted to contain energetically functional mitochondria, active in transcription of their DNA. In contrast, oocytes are predicted to obtain ATP by fermentation, by anaerobic respiration, or by import from neighbouring somatic cells [70,71]. This hypothesis (figure 2) is consistent with maternal, cytoplasmic, non-Mendelian inheritance of mitochondrially encoded phenotypic characters. The hypothesis is also consistent with sperm mitochondria, including paternal mitochondrial DNA, being targeted for autophagy and degraded soon after fertilization [72–74]. Paternal and somatic mitochondria provide energy without genetic information.

Predictions of the hypothesis described here, and depicted in figure 2, amount, so far, to proposed explanations of existing knowledge. Nevertheless, the hypothesis makes novel predictions, and a number of experimental and observational studies have the capacity to yield results that will disprove it. Here we describe the results of novel experiments designed to reveal whether oocyte mitochondria satisfy one major requirement of the hypothesis outlined in figure 2: are oocyte mitochondria transcriptionally silent, morphologically distinct and functionally quiescent?

## 5. Experimental system: the moon jellyfish, *Aurelia aurita*

The genus *Aurelia* belongs to the class Scyphozoa within the phylum Cnidaria [75]. Cnidaria are an ancient animal phylum, with the palaeontological record [76] and evolutionary inference [77] suggesting an origin prior to the Middle Cambrian period [78,79], or perhaps even during the late Proterozoic era, up to 700 million years ago [80], and before the emergence of the Bilateria [81]. Cnidaria are simple organisms consisting of an endoderm and ectoderm, between which is a largely acellular and watery ‘mesogloea’ that defines their gelatinous body. The Scyphozoa, or ‘true’ jellyfish, exhibit alternation of generations between a sexually reproductive phase, the medusa, and an asexual phase, the polyp [82]. The medusae are typically dioecious.

The genus *Aurelia* is found throughout the world's oceans [83], with *A. aurita* a very typical scyphozoan in terms of reproduction and development [84]. In wild populations, most medusae live for less than a year, dying following gamete release [84]. Horseshoe-shaped gonads are located within the gastric cavity. Fertilization is external, after which fertilized eggs transfer into brood sacs on oral arms hanging down below the umbrella where they develop into ciliated planula larvae. The mitochondrial genome of *A. aurita* is composed of 13 energy transduction protein-coding genes, small and large subunit rRNAs, and methionine and tryptophan tRNAs [85]. As one of the most ancient dioecious metazoan ancestors alive, jellyfish can be considered an ideal model organism of choice for studying evolutionarily conserved elemental features, which may span the entire animal kingdom.

## 6. Mitochondrial transcription

**Figure 3.**
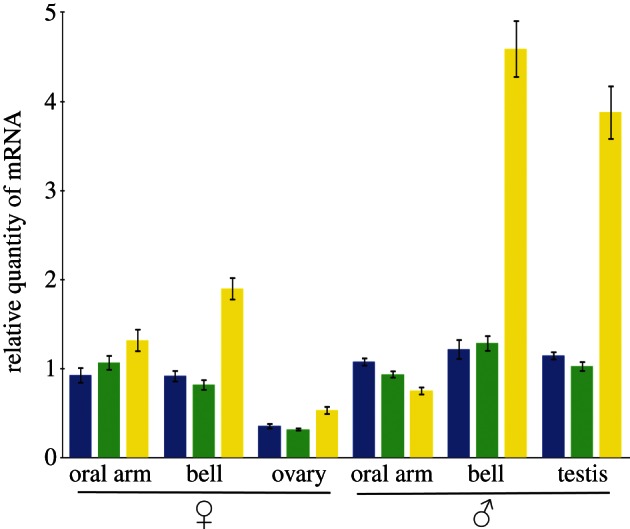
Relative quantities of mRNA from the mitochondrial genes *nad1* (blue), *cob* (green) and *cox1* (yellow) in different tissue samples from male and female medusae of the jellyfish, *Aurelia aurita*. mRNA quantities are colour coded according to the gene transcribed, as indicated also in figure 1. Mitochondrial mRNA quantities are expressed relative to those from the nuclear gene α-tubulin, and the ratio then expressed relative to the corresponding value for oral arm somatic tissue. Error bars stand for ±s.e.m. of three biological replicates. *P* < 0.05.

Mitochondrial DNA transcription is a requirement for oxidative phosphorylation *in vivo*. We performed qRT-PCR analysis to estimate the relative quantity of mitochondrial transcript of one polypeptide subunit from each of the three proton-motive respiratory chain complexes outlined schematically in figure 1. Figure 3 shows the relative quantity of mRNA from the genes *nad1*, *cob* and *cox1* in somatic tissue samples from the bell and oral arm of male and female medusae, the motile, reproductive phase, and from the female and male gonads—ovary and testis. For ovary and testis samples, a mixed population of gamete cells and surrounding diploid cells were pooled together. For all three genes, mRNA is lowest in ovary, while testis mRNA quantities are close to those of somatic tissue, notably the male bell. In males, the bell, with a high proportion of contractile tissue, appears to have the most mRNA for all three mitochondrial genes, and especially for the *cox1* subunit of cytochrome oxidase, the terminal electron acceptor for aerobic respiration. Similarly, sperm mitochondria seem to have higher amounts of *cox1* transcripts. This result fits with the assumption that oxygen supply may be a limiting factor for contractions of the bell and sperm flagellar movement. Bell contraction provides propulsion and, together with osmoconformation, [86] maintains position in the water column.

## 7. Mitochondrial ultrastructure

**Figure 4.**
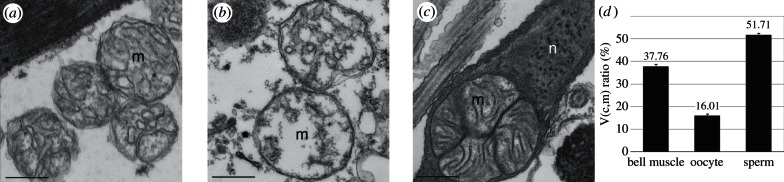
Transmission electron micrographs of *A. aurita* tissue samples. Differences in mitochondrial morphology are observed in cross-sections through (*a*) female bell, (*b*) oocyte and (*c*) spermatozoon. Mitochondria (m), nucleus (n). Scale bars, 500 nm. Stereological analysis of the morphological variations amongst the three samples is shown in (*d*). Error bars represent s.e.m, *P* < 0.01.

Figure 4*a* shows a transmission electron micrograph of a thin section of cells of a female *A. aurita* bell. Mitochondria (m) are seen to possess characteristic morphology, with an internal network of cristae; invaginations of the inner membrane into the mitochondrial matrix; and the sites of respiratory electron transport and ATP synthesis. The male bell is similar in ultrastructure (results not shown). Figure 4*b* shows the corresponding electron micrograph of an *A. aurita* oocyte; oocyte mitochondria appear slightly larger and morphologically less complex than in the bell (figure 4*a*). Figure 4*c* shows the head of an *A. aurita* spermatozoan in which the nucleus (n) lies adjacent to large, morphologically complex mitochondria (m) with numerous cristae. The contractile filaments of the flagellum consume ATP for the mechanochemical motor driving sperm motility and are in close proximity to the fully differentiated mitochondria, which can be assumed to function primarily as electrochemical fuel cells to supply the required ATP. In order to quantify the morphological variations seen in these tissues (figure 4*a–c*), we performed stereological analysis of jellyfish mitochondria of bell muscle, oocyte and sperm, as shown in figure 4*d*.

## 8. Mitochondrial membrane potential

The intermediate that couples electron transport with ATP synthesis-hydrolysis in mitochondrial energy transduction is the proton-motive force [87], a chemiosmotic gradient of electrical potential across the mitochondrial inner membrane, typically of −180 mV [88].

**Figure 5.**
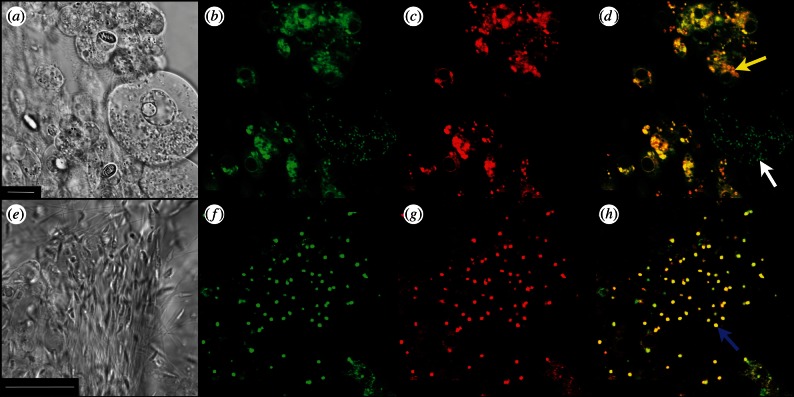
Mitochondrial membrane potential in male and female gonad samples of live *A. aurita*. Confocal light microscopy bright field view (*a,e*). Scale bars, (*a*) 10 µm; (*e*) 25 µm. Mitotracker Green FM (ex/em: 488/520 nm) detects the presence of mitochondria regardless of its activity levels (*b,f*). Conversely, Mitotracker Red FM (ex/em: 581/644 nm) is imported into active mitochondria proportionally to their membrane potential (*c,g*). Overlay images are shown in (*d,h*). Yellow arrow indicates the female diploid cell mitochondria, white arrow indicates oocyte mitochondria and the blue arrow indicates sperm mitochondria.

Confocal microscopy was used to visualize the activity of mitochondria in gonad tissue of male and female jellyfish. Figure 5*a* shows the bright field image of *Aurelia* ovarian tissue, with a scale bar of 10 µm. Figure 5*b* shows that mitochondria appear to be green because of their uptake of the fluorescent dye Mitotracker Green FM. Comparison of figure 5*a* and figure 5*b* shows that mitochondria are present, but less conspicuous, in the oocyte to the right of the picture than they are in the smaller, neighbouring, diploid cells. Figure 5*c* shows the same view of a sample of *Aurelia* ovarian tissue, this time visualized with Mitotracker Red FM, which reports specifically on the presence of the membrane potential. In surrounding diploid cells, the pattern of mitochondrial distribution seen with Mitotracker Red (figure 5*c*) agrees with that seen as the green colour of Mitotracker Green (figure 5*b*). However, and in contrast, oocyte mitochondria are unstained by Mitotracker Red (figure 5*c*), suggesting that oocyte mitochondria sustain little or no proton flux driven by membrane potential. Figure 5*d* is an overlay of the images in figure 5*b,c*, where the red and green colours of Mitotracker combine to show active mitochondria in yellow or orange. Mitochondria with low or absent membrane potential remain green, as Mitotracker Green does not respond to membrane potential. This technique has been previously described in [89].

Figure 5*e–h* presents results obtained with *Aurelia* sperm cells. Figure 5*e* is the bright field view of *Aurelia* sperm, with a 25 µm scale bar. Figure 5*f* shows the same cells visualized with Mitotracker Green, which specifically reveals a single mitochondrion lying posterior to the nucleus of each cell, as also seen by electron microscopy in figure 4*c*. Figure 5*g*, with the membrane potential-reporting Mitotracker Red, shows a picture essentially identical to that in figure 5*b*, but with green replaced by red—clearly all *Aurelia* sperm mitochondria carry a membrane potential, consistent with their primary role in ATP synthesis, serving sperm motility. Figure 5*h* presents the overlay of figure 5*b,c*, and all sperm mitochondria fully in the plane of focus are seen in yellow.

The results shown in figure 5 again correlate with the conclusion from transcription (figure 3) and ultrastructure (figure 4)—oocyte mitochondria (figure 5*a–d*) are specifically deficient in respiratory electron transport and oxidative phosphorylation, while sperm mitochondria, in contrast, are energetically fully functional (figure 5*e–h*).

## 9. Production of ROS

**Figure 6.**
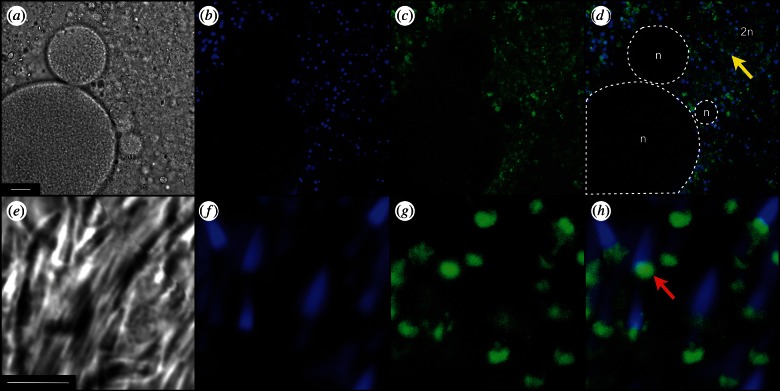
ROS content in male and female gonad samples of live *A. aurita*. Confocal light microscopy bright field view (*a,e*). Scale bars, (*a*) 25 µm; (*e*) 5 µm. 4',6-Diamidino-2-phenylindole dihydrochloride (DAPI) (ex/em: 350/450 nm) detects the presence of nuclear DNA (*b,f*). 2′,7′-Dichlorodihydrofluorescein diacetate (H_2_DCF-DA) (ex/em: 488/520 nm) imported into cells undergoes oxidation in the presence of ROS and emits green fluorescence (*c,g*). Overlay images are shown in (*d,h*). Yellow arrow indicates mitochondria in a female diploid cell, in the region labelled as ‘2n’, white dashed lines delimit oocytes, also labelled as ‘n’, and the red arrow indicates sperm mitochondria.

Figure 6*a* again shows *Aurelia* ovary in bright field light microscopy. Figure 6*b* shows the same tissue where the blue colour arises from 4',6-diamidino-2-phenylindole dihydrochloride (DAPI), a specific stain for DNA. Figure 6*c* shows green fluorescence from the oxidized form of 2′,7′-dichlorodihydrofluorescein diacetate (H_2_DCF-DA), reporting on production of ROS [90]. Figure 6*d* is an overlay of figure 6*b,c*, and suggests that oocytes (within white dashed lines) generate little or no ROS. Conversely, surrounding gonad diploid cells seem to produce enough ROS to oxidize H_2_DCF-DA, which subsequently emits the observed green fluorescence at the 520 nm range.

Figure 6*e* shows *Aurelia* sperm in bright field. Figure 6*f* shows the blue colour from sperm carrying DNA-staining DAPI. Figure 6*g* shows active production on ROS by H_2_DCF-DA in all the sperm seen in figure 6*e,f*. Figure 6*h* is an overlay, showing the blue of DAPI in the nucleus of each sperm cell anterior to the green fluorescence reporting production of ROS in the large active mitochondria. These results, from confocal light microscopy, are consistent with the sperm ultrastructure seen, by electron microscopy, in figure 4*c*.

## 10. Conclusion: oocyte mitochondria are quiescent

The results presented here provide independent lines of evidence concerning four ways in which oocyte mitochondria are distinct from sperm mitochondria and from mitochondria of somatic cells in *A. aurita*.

*Aurelia aurita* oocyte mitochondria are distinct in:

— showing decreased transcript levels of three mitochondrial genes encoding subunits of respiratory electron transport chain complexes (figure 3);
— having a simple membrane structure, without extensive internal cristae (figure 4);
— having greatly decreased capacity to accumulate the membrane potential-reporting dye Mitotracker Red (figure 5); and
— showing decreased ROS levels as measured by the ROS indicator H_2_DCF-DA (figure 6).

These are central predictions of the hypothesis presented in figure 2: oocyte mitochondria serve a primary function as genetically repressed, quiescent, inactive templates for faithful transmission of mitochondrial DNA into the next generation. It is predicted that, in other animal species, oocyte mitochondria do not accumulate mutations associated with ageing and its allied degenerative diseases. Decreased mitochondrial DNA transcription in oocytes has been noted in other animal species [91], including mammals [92–94].

## 11. Implications: the Weismann barrier and continuity of the mitochondrial germ-plasm

**Figure 7.**
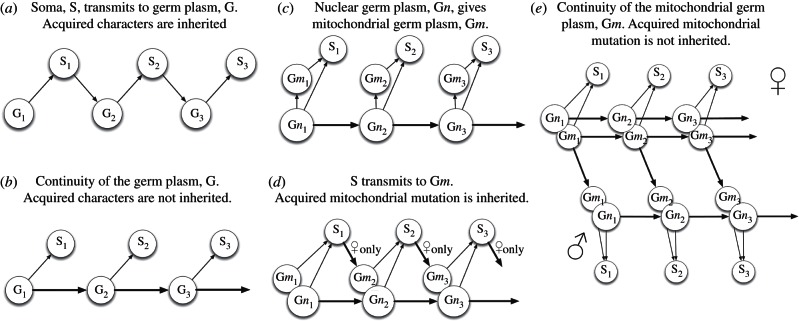
Weismann's concept of the continuity of the germ-plasm [95] extended to mitochondria. G is Germ-plasm; the hereditary material. S is soma; the phenotype. Today, following Crick [96], we regard DNA as the molecular basis of G, and protein, broadly, as the molecular foundation of S. Arrows indicate direction of flow of information. Subscripts indicate generation number. Thick arrows indicate transmission of genetic information, including mutation. (*a*) The pre-Weismann view, associated with Lamarck, that S derives from G, and G derives from S. By this view, acquired characters are inherited. (*b*) Weismann's hypothesis. S derives from G, and G derives only from previous G. Selection acts on S, determining only whether G is passed on to the next generation. Information does not flow from S to G: this restriction can be termed the Weismann Barrier. (*c*) Autogeny of cells, including the mitochondria and mitochondrial DNA, from a single genome [97]; mitochondria are effectively part of S in (*b*). (*d*) A conventional modern view of combined mitochondrial (m) and nuclear (n) inheritance. The Weismann barrier is broken because acquired characters of mitochondria, for example, ROS-induced mutation, convey heritable information, in the mitochondrial genome, from S to G, and, for some reason, only in females. If mitochondria are the cause of ageing, then ageing should be inherited, and accumulate in successive generations [98]. (*e*) Mitochondrial division of labour between male and female (see figure 2) preserves the Weismann barrier for mitochondrial as well as nuclear inheritance. ♀, Female: mitochondrial G and nuclear G pass directly between generations. S determines only whether G transmits at all, and has no informational input into mitochondrial DNA. ♂, Male: mitochondrial G exists only to contribute to S, and is never a genetic template. Instead, mitochondrial G is maternally inherited in the cytoplasm of oocytes, where it is carried by quiescent mitochondria and thus transmitted independently of S.

Contrary to a common assumption of the heritability of characters acquired by an individual within its lifetime, Weismann proposed, in the nineteenth century, a doctrine, or ‘dogma’, of the continuity of the germ-plasm [95]. In schematic outline, figure 7*b* depicts Weismann's hypothesis and figure 7*a* the earlier, contrary view, propounded by Lamarck, in which the germ line by some means encapsulates and transmits characteristics of the soma, including those acquired from physiological responses to environmental challenges [17,99]. Today Weismann's hypothesis can be restated in genetic terms: the phenotype arises from the genotype while the genotype arises from a pre-existing genotype. The phenotype is the outward expression of the genotype; it serves as a vehicle for environmental interactions that determine whether the genotype survives to be passed on to the next generation. Mendelian genetics and the Darwin–Wallace theory of evolution by natural selection are together broadly consistent with Weismann and count against Lamarck. The ‘Central Dogma’ of molecular biology [96], the one-way flow of information from nucleotide sequence to amino acid sequence, lays a foundation for the existence of the ‘Weismann barrier’, prohibiting transfer of genetic information from protein to nucleotide sequence, from phenotype to genotype, or, in Weismannian terminology, from soma to germ-plasm. In this context, mitochondrial DNA has been viewed in different ways, outlined in figure 7*c,d*. Figure 7*d* may represent a current consensus. In figure 7, mitochondrial mutation is counted as an acquired character because it occurs within an individual lifetime, may have immediate effects, and varies in frequency in response to physiological and environmental change.

The hypothesis in figure 2 and supported by evidence presented here (figures 3–6) proposes that Weismann's rule applies equally to mitochondrial inheritance, as outlined in figure 7*e*. In this view, mitochondrial energy transduction is adaptable and physiologically responsive to environmental change, while the continuity of mitochondrial DNA is secured by a separate and self-contained life cycle of template mitochondria, themselves unexposed directly, and as a matter of course, to physiological constraints that have effects on mitochondrial DNA sequence. If the hypothesis depicted in figure 2 is correct, then there is no need to postulate the existence of a process for distillation of mitochondrial phenotype into genotype, or for ‘purifying’ selection of mitochondrial that are, by some means, deemed fit for transmission from among a heteroplasmic population [100,101]. This is not to forbid the concepts of a bottleneck in oogenesis [102] or of mito-nuclear co-adaptation [103,104]—clearly the chimeric nature of key respiratory chain complexes (figure 1) will mean that different combinations of nuclear genes can be more compatible, or less, with safe and efficient mitochondrial energy transduction. Therefore, natural selection will act on the consequences of favourable or unfavourable combinations of nuclear with mitochondrial genes. The hypothesis in figure 2, however, removes the need to suppose that mito-nuclear interactions act with foresight of phenotypic consequences by anticipating the selective advantage of co-location of any specific mitochondrial DNA sequence with the respiratory apparatus that it partly encodes.

There are occasional variants from the maternal mode of mitochondrial inheritance. For example, in the mussel, *Mytilus,* there is biparental mitochondrial inheritance by sons, while daughters acquire mitochondria by uniparental, maternal inheritance alone [105]. More generally, the Weismann barrier applied to mitochondrial inheritance (figure 7*e*) forbids reversion of energy-transducing mitochondria into quiescent, genetic templates. We intend to search for this reversion as a stringent test, and potential disproof, of the hypothesis in figure 2. Until there is evidence to the contrary, we propose that female germ lines maintain, carry and transmit quiescent mitochondria as the cytoplasmic genetic templates from which all other mitochondria ultimately derive.

## Appendix. Material and methods

### A.1. Jellyfish

Live male and female medusae were collected from Horsea Lake, Hampshire, UK on 28 August 2012 and 12 November 2012. Fresh tissue samples were taken from live specimens that had been maintained for up to 7 days in aerated artificial seawater diluted with distilled water to give a final salinity of 25, corresponding to the original brackish lake water.

### A.2. RNA isolation and qRT-PCR analysis

Total RNA from dissected *A. aurita* tissues was isolated using TRI reagent (Ambion), treated with DNase I (New England Biolabs) for 10 min at 37 °C and re-purified using Pure LinkTM RNA Mini Kit (Ambion), all according to the manufacturers’ instructions. A Chromo4 real-time detector (Bio-Rad), and Brilliant III Ultra-Fast SYBR Green qRT-PCR (Agilent Technologies) were used for the reactions. All mitochondrial messenger RNA quantities were normalized against the nuclear gene α-tubulin. A second normalization was done using oral arms from both sexes as the reference. Primers used in this study were: nad1-F: 5′-GGAGTGGTTTGGGACAGTCA-3′, nad1-R: 5′-TCGAGGAAAGGGCCAAATGT-3′, cob-F: 5′-AACATGGGCAGCACCTAGTC-3′, cob-R: 5′-ACTTGGTTTCAGCTGTTCCCT-3′, cox1-F: 5′-ATGGTGGGAACTGCCTTCAG-3′, cox1-R: 5′-TTGATCGTCCCCCAACATGG-3′, α-tubulin-F: 5′-CAGCACCCCAAATCTCCACT-3′ and α-tubulin-R: 5′-ACCATGAAAGCGCAGTCAGA-3′. Three technical replicates for each biological replicate were averaged at the beginning, and three biological replicates were used to generate the error bars. *C*(*t*) values were analysed using qBase PLUS2 software (Biogazelle).

### A.3. Transmission electron microscopy

For transmission electron microscopy, specimens were fixed overnight in 2.5% (v/v) glutaraldehyde in 0.1 M cacodylate buffer (pH 7.3) supplemented with 0.14 M NaCl and post-fixed in 1% (w/v) osmium tetroxide in 0.1 M cacodylate buffer (pH 7.3) for 1.5 hours. Samples were then dehydrated through a graded ethanol series, equilibrated with propylene oxide before infiltration with TAAB SPURR resin and polymerized at 70 °C for 24 h. Ultrathin sections (70–90 nm) were prepared using a Reichert-Jung Ultracut E ultramicrotome, mounted on 150 mesh copper grids, contrasted using uranyl acetate and lead citrate and examined on an FEI Tecnai 12 transmission microscope operated at 120 kV. Images were acquired with an AMT 16000M digital camera. Stereological analysis was carried out as described in [106]. Thirty mitochondria from each group (bell muscle, oocyte and sperm) were used for the analysis. The combined point counting grid used for this study was composed of two sets of points of different densities on the same grid; nine fine points per coarse point. The volume of reference (mitochondria) was estimated by counting the number of coarse points that hit the reference space and multiplied by nine. The volume of the particular phase (cristae) was estimated by counting the number of fine and coarse points that hit the cristae. Student's *t*-test analysis was performed to determine the statistical significance.

### A.4. Confocal microscopy

For mitochondrial membrane potential analysis, freshly dissected tissues of *A. aurita* were equilibrated for approximately 30 min at room temperature in solutions of Mitotracker Red FM (Life Technologies) or Mitotracker Green FM (Life Technologies) dissolved in PBS to give a final concentration of 500 nM. Three washes in pure PBS were used to remove the probe excess. For ROS detection, we followed the method described in [90] with minor modifications. Freshly dissected tissues were equilibrated in 10 µM 2′,7′-dichlorodihydrofluorescein diacetate (H_2_DCF-DA) solution, also containing DAPI (Sigma) (1 µM final concentration) in room temperature PBS for 30 min, followed by three PBS washes. Microscopy was performed using a Leica SP5 confocal microscope with an 8-bit camera (Leica Microsystems). Excitation and emission wavelengths were selected for the different fluorescent dyes as follows: DAPI: 350/450 to 480 nm, H_2_DCF-DA: 488/520 to 550 nm, Mitotracker Green: 488/500 to 530 nm, and Mitotracker Red: 581/620 to 650 nm. Imaging was performed with a 63× lens, and laser output was kept under 15% of maximal power. Leica LAS AF software was used to acquire and analyse the images.

## References

[1] Mitchell P 1961 Coupling of phosphorylation to electron and hydrogen transfer by a chemi-osmotic type of mechanism. Nature 191, 144–148 10.1038/191144a0 (doi:10.1038/191144a0) 13771349

[2] Roodyn DB Wilkie D 1968 The biogenesis of mitochondria. London: Methuen

[3] Haslbrunner E Tuppy H Schatz G 1964 Deoxyribonucleic acid associated with yeast mitochondria. Biochem. Biophys. Res. Commun. 15, 127–132 10.1016/0006-291X(64)90311-0 (doi:10.1016/0006-291X(64)90311-0) 26410904

[4] Horner DS Hirt RP Kilvington S Lloyd D Embley TM 1996 Molecular data suggest an early acquisition of the mitochondrion endosymbiont. Proc. R. Soc. Lond. B 263, 1053–1059 10.1098/rspb.1996.0155 (doi:10.1098/rspb.1996.0155) 8805838

[5] Martin W Müller M 2007 Origin of mitochondria and hydrogenosomes. Berlin, Heidelberg: Springer

[6] Yang D Oyaizu Y Oyaizu H Olsen GJ Woese CR 1985 Mitochondrial origins. Proc. Natl Acad. Sci. USA 82, 4443–4447 10.1073/pnas.82.13.4443 (doi:10.1073/pnas.82.13.4443) 3892535 PMC391117

[7] Sagan L 1967 On the origin of mitosing cells. J. Theor. Biol. 14, 255–274 10.1016/0022-5193(67)90079-3 (doi:10.1016/0022-5193(67)90079-3) 11541392

[8] Nass MM Nass S 1963 Intramitochondrial fibers with DNA characteristics. I. Fixation and electron staining reactions. J. Cell Biol. 19, 593–611 10.1083/jcb.19.3.593 (doi:10.1083/jcb.19.3.593) 14086138 PMC2106331

[9] Luck DJL Reich E 1964 DNA in mitochondria of *Neurospora crassa*. Proc. Natl Acad. Sci. USA 52, 931–938 10.1073/pnas.52.4.931 (doi:10.1073/pnas.52.4.931) 14224397 PMC300375

[10] Anderson S 1981 Sequence and organization of the human mitochondrial genome. Nature 290, 457–465 10.1038/290457a0 (doi:10.1038/290457a0) 7219534

[11] Pesole G Allen JF Lane N Martin W Rand DM Schatz G Saccone C 2012 The neglected genome. EMBO Rep. 13, 473–474 10.1038/embor.2012.57 (doi:10.1038/embor.2012.57) 22555611 PMC3367242

[12] Claros MG Perea J Shu Y Samatey FA Popot JL Jacq C 1995 Limitations to *in vivo* import of hydrophobic proteins into yeast mitochondria: the case of a cytoplasmically synthesized apocytochrome *b*. Eur. J. Biochem. 228, 762–771 10.1111/j.1432-1033.1995.0762m.x (doi:10.1111/j.1432-1033.1995.0762m.x) 7737175

[13] Koehler CM Merchant S Schatz G 1999 How membrane proteins travel across the mitochondrial intermembrane space. Trends Biochem. Sci. 24, 428–432 10.1016/S0968-0004(99)01462-0 (doi:10.1016/S0968-0004(99)01462-0) 10542408

[14] Allen JF 2003 The function of genomes in bioenergetic organelles. Phil. Trans. R. Soc. Lond. B 358, 19–38 10.1098/rstb.2002.1191 (doi:10.1098/rstb.2002.1191) 12594916 PMC1693096

[15] Camus M Clancy DJ Dowling DK 2012 Mitochondria, maternal inheritance, and male aging. Curr. Biol. 22, 1–5 10.1016/j.cub.2011.12.009 (doi:10.1016/j.cub.2011.12.009) 22863313

[16] Wallace DC 2007 Why do we still have a maternally inherited mitochondrial DNA? Insights from evolutionary medicine. Annu. Rev. Biochem. 76, 781–821 10.1146/annurev.biochem.76.081205.150955 (doi:10.1146/annurev.biochem.76.081205.150955) 17506638

[17] Whitehouse HLK 1969 Towards an understanding of the mechanism of heredity, 2nd edn London: Edward Arnold (Publishers) Ltd

[18] Allen JF de Paula WB Puthiyaveetil S Nield J 2011 A structural phylogenetic map for chloroplast photosynthesis. Trends Plant. Sci. 16, 645–655 10.1016/j.tplants.2011.10.004 (doi:10.1016/j.tplants.2011.10.004) 22093371

[19] Allen JF 1993 Control of gene-expression by redox potential and the requirement for chloroplast and mitochondrial genomes. J. Theor. Biol. 165, 609–631 10.1006/jtbi.1993.1210 (doi:10.1006/jtbi.1993.1210) 8114509

[20] León-Avila G Tovar J 2004 Mitosomes of *Entamoeba histolytica* are abundant mitochondrion-related remnant organelles that lack a detectable organellar genome. Microbiology 150, 1245–1250 10.1099/mic.0.26923-0 (doi:10.1099/mic.0.26923-0) 15133087

[21] Tovar J Fischer A Clark CG 1999 The mitosome, a novel organelle related to mitochondria in the amitochondrial parasite *Entamoeba histolytica*. Mol. Microbiol. 32, 1013–1021 10.1046/j.1365-2958.1999.01414.x (doi:10.1046/j.1365-2958.1999.01414.x) 10361303

[22] Van Der Giezen M 2009 Hydrogenosomes and mitosomes: conservation and evolution of functions. J. Eukaryot. Microbiol. 56, 221–231 10.1111/j.1550-7408.2009.00407.x (doi:10.1111/j.1550-7408.2009.00407.x) 19527349

[23] Williams BAP Hirt RP Lucocq JM Embley TM 2002 A mitochondrial remnant in the microsporidian *Trachipleistophora hominis*. Nature 418, 865–869 10.1038/nature00949 (doi:10.1038/nature00949) 12192407

[24] Allen JF 2003 Why chloroplasts and mitochondria contain genomes. Comp. Funct. Genomics 4, 31–36 10.1002/cfg.245 (doi:10.1002/cfg.245) 18629105 PMC2447392

[25] Daley DO Clifton R Whelan J 2002 Intracellular gene transfer: reduced hydrophobicity facilitates gene transfer for subunit 2 of cytochrome c oxidase. Proc. Natl Acad. Sci. USA 99, 10 510–10 515 10.1073/pnas.122354399 (doi:10.1073/pnas.122354399) 12142462 PMC124958

[26] Martin W Hoffmeister M Rotte C Henze K 2001 An overview of endosymbiotic models for the origins of eukaryotes, their ATP-producing organelles (mitochondria and hydrogenosomes), and their heterotrophic lifestyle. Biol. Chem. 382, 1521–1539 10.1515/BC.2001.187 (10.1515/BC.2001.187) 11767942

[27] von Heijne G 1986 Why mitochondria need a genome. FEBS Lett. 198, 1–4 10.1016/0014-5793(86)81172-3 (doi:10.1016/0014-5793(86)81172-3) 3514271

[28] Race HL Herrmann RG Martin W 1999 Why have organelles retained genomes? Trends Genet. 15, 364–370 10.1016/S0168-9525(99)01766-7 (doi:10.1016/S0168-9525(99)01766-7) 10461205

[29] Barbrook AC Howe CJ Purton S 2006 Why are plastid genomes retained in non-photosynthetic organisms? Trends Plant Sci. 11, 101–108 10.1016/j.tplants.2005.12.004 (doi:10.1016/j.tplants.2005.12.004) 16406301

[30] Pfannschmidt T Nilsson A Allen JF 1999 Photosynthetic control of chloroplast gene expression. Nature 397, 625–628 10.1038/17624 (doi:10.1038/17624)

[31] Puthiyaveetil S Ibrahim IM Jelicic B Tomasic A Fulgosi H Allen JF 2010 Transcriptional control of photosynthesis genes: the evolutionarily conserved regulatory mechanism in plastid genome function. Genome Biol. Evol. 2, 888–896 10.1093/evq073 (doi:10.1093/evq073) 21071627 PMC3012001

[32] Allen CA Håkansson G Allen JF 1995 Redox conditions specify the proteins synthesized by isolated- chloroplasts and mitochondria. Redox Rep. 1, 119–123 10.1080/13510002.1995.11746969 27405554

[33] de Paula WBM Allen JF van der Giezen M 2012 Mitochondria, hydrogenosomes and mitosomes in relation to the CoRR hypothesis for genome function and evolution. In Organelle genetics (ed. Bullerwell CE), pp. 105–119 Berlin, Heidelberg: Springer

[34] Puthiyaveetil S 2008 The ancestral symbiont sensor kinase CSK links photosynthesis with gene expression in chloroplasts. Proc. Natl Acad. Sci. USA 105, 10 061–10 066 10.1073/pnas.0803928105 (doi:10.1073/pnas.0803928105) PMC2474565 18632566

[35] Puthiyaveetil S Ibrahim IM Allen JF 2013 Evolutionary rewiring: a modified prokaryotic gene regulatory pathway in chloroplasts. Phil. Trans. R. Soc. B 367, 20120260 10.1098/rstb20120260 (doi:10.1098/rstb20120260). 23754813 PMC3685462

[36] Allen JF Raven JA 1996 Free-radical-induced mutation vs redox regulation: costs and benefits of genes in organelles. J. Mol. Evol. 42, 482–492 10.1007/BF02352278 (doi:10.1007/BF02352278) 8662000

[37] Chance B Sies H Boveris A 1979 Hydroperoxide metabolism in mammalian organs. Physiol. Rev. 59, 527–605 37532 10.1152/physrev.1979.59.3.527

[38] Chen Q Vazquez EJ Moghaddas S Hoppel CL Lesnefsky EJ 2003 Production of reactive oxygen species by mitochondria: central role of complex III. J. Biol. Chem. 278, 36 027–36 031 10.1074/jbc.M304854200 (doi:10.1074/jbc.M304854200) 12840017

[39] Ohnishi ST Shinzawa-Itoh K Ohta K Yoshikawa S Ohnishi T 2010 New insights into the superoxide generation sites in bovine heart NADH-ubiquinone oxidoreductase (complex I) The significance of protein-associated ubiquinone and the dynamic shifting of generation sites between semiflavin and semiquinone radicals. Bba-Bioenergetics 1797, 1901–1909 10.1016/j.bbabio.2010.05.012 (doi:10.1016/j.bbabio.2010.05.012) 20513438

[40] Kussmaul L Hirst J 2006 The mechanism of superoxide production by NADH: ubiquinone oxidoreductase (complex I) from bovine heart mitochondria. Proc. Natl Acad. Sci. USA 103, 7607–7612 10.1073/pnas.0510977103 (doi:10.1073/pnas.0510977103) 16682634 PMC1472492

[41] McCord JM Fridovich I 1969 Superoxide dismutase. An enzymic function for erythrocuprein (hemocuprein). J. Biol. Chem. 244, 6049–6055 5389100

[42] Li Y 1995 Dilated cardiomyopathy and neonatal lethality in mutant mice lacking manganese superoxide dismutase. Nat. Genet. 11, 376–381 10.1038/ng1295-376 (doi:10.1038/ng1295-376) 7493016

[43] Melov S Schneider JA Day BJ Hinerfeld D Coskun P Mirra SS Crapo JD Wallace DC 1998 A novel neurological phenotype in mice lacking mitochondrial manganese superoxide dismutase. Nat. Genet. 18, 159–163 10.1038/ng0298-159 (doi:10.1038/ng0298-159) 9462746

[44] Taufer M Peres A de Andrade VM de Oliveira G Sa G do Canto ME dos Santos AR Bauer ME da Cruz IB 2005 Is the Val16Ala manganese superoxide dismutase polymorphism associated with the aging process? J. Gerontol. A Biol. Sci. Med. Sci. 60, 432–438 10.1093/gerona/60.4.432 (doi:10.1093/gerona/60.4.432) 15933380

[45] Treiber N 2011 Accelerated aging phenotype in mice with conditional deficiency for mitochondrial superoxide dismutase in the connective tissue. Aging Cell 10, 239–254 10.1111/j.1474-9726.2010.00658.x (doi:10.1111/j.1474-9726.2010.00658.x) 21108731

[46] Pessayre D Berson A Fromenty B Mansouri A 2001 Mitochondria in steatohepatitis. Semin. Liver Dis. 21, 57–69 10.1055/s-2001-12929 (doi:10.1055/s-2001-12929) 11296697

[47] Berlett BS Stadtman ER 1997 Protein oxidation in aging, disease, and oxidative stress. J. Biol. Chem. 272, 20 313–20 316 10.1074/jbc.272.1.20 (doi:10.1074/jbc.272.1.20) 9252331

[48] Cooke MS Evans MD Dizdaroglu M Lunec J 2003 Oxidative DNA damage: mechanisms, mutation, and disease. FASEB J. 17, 1195–1214 10.1096/fj.02-0752rev (doi:10.1096/fj.02-0752rev) 12832285

[49] Ames BN Shigenaga MK Hagen TM 1993 Oxidants, antioxidants, and the degenerative diseases of aging. Proc. Natl Acad. Sci. USA 90, 7915–7922 10.1073/pnas.90.17.7915 (doi:10.1073/pnas.90.17.7915) 8367443 PMC47258

[50] Ames BN Shigenaga MK Hagen TM 1995 Mitochondrial decay in aging. Biochim. Biophys. Acta 1271, 165–170 10.1016/0925-4439(95)00024-X (doi:10.1016/0925-4439(95)00024-X) 7599204

[51] Harman D 1972 The biologic clock: the mitochondria? J. Am. Geriatr. Soc. 20, 145–147 5016631 10.1111/j.1532-5415.1972.tb00787.x

[52] Harman D 1992 Free radical theory of aging. Mutat. Res. 275, 257–266 10.1016/0921-8734(92)90030-S (doi:10.1016/0921-8734(92)90030-S) 1383768

[53] Shigenaga MK Hagen TM Ames BN 1994 Oxidative damage and mitochondrial decay in aging. Proc. Natl Acad. Sci. USA 91, 10 771–10 778 10.1073/pnas.91.23.10771 (doi:10.1073/pnas.91.23.10771) PMC45108 7971961

[54] Petros JA 2005 mtDNA mutations increase tumorigenicity in prostate cancer. Proc. Natl Acad. Sci. USA 102, 719–724 10.1073/pnas.0408894102 (doi:10.1073/pnas.0408894102) 15647368 PMC545582

[55] Afanas'ev I 2010 Signaling and damaging functions of free radicals in aging–free radical theory, hormesis, and TOR. Aging Dis. 1, 75–88 22396858 PMC3295029

[56] Jacobs HT 2003 The mitochondrial theory of aging: dead or alive? Aging Cell 2, 11–17 10.1046/j.1474-9728.2003.00032.x (doi:10.1046/j.1474-9728.2003.00032.x) 12882330

[57] Park CB Larsson NG 2011 Mitochondrial DNA mutations in disease and aging. J. Cell Biol. 193, 809–818 10.1083/jcb.201010024 (doi:10.1083/jcb.201010024) 21606204 PMC3105550

[58] Kujoth GC 2005 Mitochondrial DNA mutations, oxidative stress, and apoptosis in mammalian aging. Science 309, 481–484 10.1126/science.1112125 (doi:10.1126/science.1112125) 16020738

[59] Trifunovic A 2004 Premature ageing in mice expressing defective mitochondrial DNA polymerase. Nature 429, 417–423 10.1038/nature02517 (doi:10.1038/nature02517) 15164064

[60] Trifunovic A 2005 Somatic mtDNA mutations cause aging phenotypes without affecting reactive oxygen species production. Proc. Natl Acad. Sci. USA 102, 17 993–17 998 10.1073/pnas.0508886102 (doi:10.1073/pnas.0508886102) PMC1312403 16332961

[61] Sasaki T Unno K Tahara S Shimada A Chiba Y Hoshino M Kaneko T 2008 Age-related increase of superoxide generation in the brains of mammals and birds. Aging Cell 7, 459–469 10.1111/j.1474-9726.2008.00394.x (doi:10.1111/j.1474-9726.2008.00394.x) 18419797

[62] Mendoza-Nunez VM Ruiz-Ramos M Sanchez-Rodriguez MA Retana-Ugalde R Munoz-Sanchez JL 2007 Aging-related oxidative stress in healthy humans. Tohoku J. Exp. Med. 213, 261–268 10.1620/tjem.213.261 (doi:10.1620/tjem.213.261) 17984623

[63] Miyazawa M Ishii T Yasuda K Noda S Onouchi H Hartman PS Ishii N 2009 The role of mitochondrial superoxide anion (O_2_^−^) on physiological aging in C57BL/6J mice. J. Radiat. Res. 50, 73–83 10.1269/jrr.08097 (doi:10.1269/jrr.08097) 19218782

[64] Lund DD Chu Y Miller JD Heistad DD 2009 Protective effect of extracellular superoxide dismutase on endothelial function during aging. Am. J. Physiol. Heart Circ. Physiol. 296, H1920–H1925 10.1152/ajpheart.01342.2008 (doi:10.1152/ajpheart.01342.2008) 19376805 PMC2716111

[65] Donato AJ Eskurza I Silver AE Levy AS Pierce GL Gates PE Seals DR 2007 Direct evidence of endothelial oxidative stress with aging in humans: relation to impaired endothelium-dependent dilation and upregulation of nuclear factor-κB. Circ. Res. 100, 1659–1666 10.1161/01.RES.0000269183.13937.e8 (doi:10.1161/01.RES.0000269183.13937.e8) 17478731

[66] Choksi KB Roberts LJ 2nd DeFord JH Rabek JP Papaconstantinou J 2007 Lower levels of F2-isoprostanes in serum and livers of long-lived Ames dwarf mice. Biochem. Biophys. Res. Commun. 364, 761–764 10.1016/j.bbrc.2007.10.100 (doi:10.1016/j.bbrc.2007.10.100) 17964285 PMC2238179

[67] Jacobson A Yan C Gao Q Rincon-Skinner T Rivera A Edwards J Huang A Kaley G Sun D 2007 Aging enhances pressure-induced arterial superoxide formation. Am. J. Physiol. Heart Circ. Physiol. 293, H1344–H1350 10.1152/ajpheart.00413.2007 (doi:10.1152/ajpheart.00413.2007) 17557915 PMC4536921

[68] Lener B Koziel R Pircher H Hutter E Greussing R Herndler-Brandstetter D Hermann M Unterluggauer H Jansen-Durr P 2009 The NADPH oxidase Nox4 restricts the replicative lifespan of human endothelial cells. Biochem. J. 423, 363–374 10.1042/BJ20090666 (doi:10.1042/BJ20090666) 19681754 PMC2762686

[69] Rodriguez-Manas L 2009 Endothelial dysfunction in aged humans is related with oxidative stress and vascular inflammation. Aging Cell 8, 226–238 10.1111/j.1474-9726.2009.00466.x (doi:10.1111/j.1474-9726.2009.00466.x) 19245678

[70] Allen JF 1996 Separate sexes and the mitochondrial theory of ageing. J. Theor. Biol. 180, 135–140 10.1006/jtbi.1996.0089 (doi:10.1006/jtbi.1996.0089) 8763364

[71] Allen CA van der Giezen M Allen JF 2007 Origin, function and transmission of mitochondria. In Origins of mitochondria and hydrogenosomes (eds Martin W Muller M), pp. 39–56 Berlin, Heidelberg: Springer

[72] Al Rawi S Louvet-Vallee S Djeddi A Sachse M Culetto E Hajjar C Boyd L Legouis R Galy V 2011 Postfertilization autophagy of sperm organelles prevents paternal mitochondrial DNA transmission. Science 334, 1144–1147 10.1126/science.1211878 (doi:10.1126/science.1211878) 22033522

[73] Deluca SZ O'Farrell PH 2013 Barriers to male transmission of mitochondrial DNA in sperm development. Dev. Cell 22, 660–668 10.1016/j.devcel.2011.12.021 (doi:10.1016/j.devcel.2011.12.021) 22421049 PMC3306594

[74] Sato M Sato K 2011 Degradation of paternal mitochondria by fertilization-triggered autophagy in *C. elegans* embryos. Science 334, 1141–1144 10.1126/science.1210333 (doi:10.1126/science.1210333) 21998252

[75] Collins AG Schuchert P Marques AC Jankowski T Medina M Schierwater B 2006 Medusozoan phylogeny and character evolution clarified by new large and small subunit rDNA data and an assessment of the utility of phylogenetic mixture models. Syst. Biol. 55, 97–115 10.1080/10635150500433615 (doi:10.1080/10635150500433615) 16507527

[76] Cartwright P Halgedahl SL Hendricks JR Jarrard RD Marques AC Collins AG Lieberman BS 2007 Exceptionally preserved jellyfishes from the middle Cambrian. PLoS One 2, e1121 10.3410/f.1093897.548834 (doi:10.3410/f.1093897.548834) 17971881 PMC2040521

[77] Ojimi MC Hidaka M 2010 Comparison of telomere length among different life cycle stages of the jellyfish *Cassiopea andromeda*. Mar. Biol. 157, 2279–2287 10.1007/s00227-010-1495-4 (doi:10.1007/s00227-010-1495-4)

[78] Butterfield NJ 1997 Plankton ecology and the Proterozoic–Phanerozoic transition. Paleobiology 23, 247–262

[79] Condon RH 2012 Questioning the rise of gelatinous zooplankton in the world's oceans. Bioscience 62, 160–169 10.1525/bio.2012.62.2.9 (doi:10.1525/bio.2012.62.2.9)

[80] Butterfield NJ 2000 *Bangiomorpha pubescens* n. gen, n. sp.: implications for the evolution of sex, multicellularity, and the Mesoproterozoic/Neoproterozoic radiation of eukaryotes. Paleobiology 26, 386–404 10.1666/0094-8373(2000)026&lt;0386:BPNGNS&gt;2.0.CO;2 (doi:10.1666/0094-8373(2000)026&lt;0386:BPNGNS&gt;2.0.CO;2)

[81] Extavour CG Akam M 2003 Mechanisms of germ cell specification across the metazoans: epigenesis and preformation. Development 130, 5869–5884 10.1242/dev.00804 (doi:10.1242/dev.00804) 14597570

[82] Morandini AC Da Silveira FL 2001 Sexual reproduction of *Nausithoe aurea* (Scyphozoa, Coronatae) gametogenesis, egg release, embryonic development, and gastrulation. Sci. Mar. 65, 139–149

[83] Dawson MN Martin LE 2001 Geographic variation and ecological adaptation in *Aurelia* (Scyphozoa, Semaeostomeae): some implications from molecular phylogenetics. Hydrobiologia 451, 259–273 10.1023/A:1011869215330 (doi:10.1023/A:1011869215330)

[84] Lucas CH 2001 Reproduction and life history strategies of the common jellyfish, *Aurelia aurita*, in relation to its ambient environment. Hydrobiologia 451, 229–246 10.1023/A:1011836326717 (doi:10.1023/A:1011836326717)

[85] Shao ZY Graf S Chaga OY Lavrov DV 2006 Mitochondrial genome of the moon jelly *Aurelia aurita* (Cnidaria, Scyphozoa): a linear DNA molecule encoding a putative DNA-dependent DNA polymerase. Gene 381, 92–101 10.1016/j.gene.2006.06.021 (doi:10.1016/j.gene.2006.06.021) 16945488

[86] Hirst AG Lucas CH 1998 Salinity influences body weight quantification in the scyphomedusa *Aurelia aurita*: important implications for body weight determination in gelatinous zooplankton. Mar Ecol Prog Ser 165, 259–269 10.3354/meps165259 (doi:10.3354/meps165259)

[87] Mitchell P 1979 Keilins respiratory-chain concept and its chemiosmotic consequences. Science 206, 1148–1159 10.1126/science.388618 (doi:10.1126/science.388618) 388618

[88] Rich P 2003 The cost of living. Nature 421, 583–583 10.1038/421583a (doi:10.1038/421583a) 12571574

[89] Pendergrass W Wolf N Poot M 2004 Efficacy of MitoTracker Green and CMXrosamine to measure changes in mitochondrial membrane potentials in living cells and tissues. Cytometry A 61, 162–169 10.1002/cyto.a.20033 (doi:10.1002/cyto.a.20033) 15382028

[90] Owusu-Ansah E Yavari A Banerjee U 2008 A protocol for *in vivo* detection of reactive oxygen species Protocol Exchange 10.1038/nprot.2008.23 (doi:10.1038/nprot.2008.23)

[91] de Paula WBM Agip A-N Missirlis F Ashworth R Vizcay-Barrena G Lucas C Allen JF Submitted. Mitochondrial function in the origin of the female germ line

[92] Hsieh RH Au HK Yeh TS Chang SJ Cheng YF Tzeng CR 2004 Decreased expression of mitochondrial genes in human unfertilized oocytes and arrested embryos. Fertil. Steril. 81 (Suppl. 1), 912–918 10.1016/j.fertnstert.2003.11.013 (doi:10.1016/j.fertnstert.2003.11.013) 15019829

[93] Santos TA Shourbagy SE John JCS 2006 Mitochondrial content reflects oocyte variability and fertilization outcome. Fertil. Steril. 85, 584–591 10.1016/j.fertnstert.2005.09.017 (doi:10.1016/j.fertnstert.2005.09.017) 16500323

[94] Wilding M Dale B Marino M diMatteo L Alviggi C Pisaturo ML Lombardi L dePlacido G 2001 Mitochondrial aggregation patterns and activity in human oocytes and preimplantation embryos. Hum. Reprod. 16, 909–917 10.1093/humrep/16.5.909 (doi:10.1093/humrep/16.5.909) 11331637

[95] Weismann A 1889 Essays upon heredity, *vols 1 and 2*. Oxford: The Clarendon Press

[96] Crick F 1970 Central dogma of molecular biology. Nature 227, 561–563 10.1038/227561a0 (doi:10.1038/227561a0) 4913914

[97] Bogorad L 1975 Evolution of organelles and eukaryotic genomes. Science 188, 891–898 10.1126/science.1138359 (doi:10.1126/science.1138359) 1138359

[98] Allen JF Allen CA 1999 A mitochondrial model for premature ageing of somatically cloned mammals. IUBMB Life 48, 369–372 10.1080/713803544 (doi:10.1080/713803544) 10632563

[99] Maynard Smith J 1966 The theory of evolution, 2nd edn London: Penguin Books

[100] Stewart JB Freyer C Elson JL Wredenberg A Cansu Z Trifunovic A Larsson NG 2008 Strong purifying selection in transmission of mammalian mitochondrial DNA. PLoS Biol. 6, e10 10.1371/journal.pbio.0060010 (doi:10.1371/journal.pbio.0060010) 18232733 PMC2214808

[101] Fan W 2008 A mouse model of mitochondrial disease reveals germline selection against severe mtDNA mutations. Science 319, 958–962 10.1126/science.1147786 (doi:10.1126/science.1147786) 18276892 PMC3049809

[102] Reynier P Chretien MF Savagner F Larcher G Rohmer V Barriere P Malthiery Y 1998 Long PCR analysis of human gamete mtDNA suggests defective mitochondrial maintenance in spermatozoa and supports the bottleneck theory for oocytes. Biochem. Biophys. Res. Commun. 252, 373–377 10.1006/bbrc.1998.9651 (doi:10.1006/bbrc.1998.9651) 9826537

[103] Hadjivasiliou Z Pomiankowski A Seymour RM Lane N 2012 Selection for mitonuclear co-adaptation could favour the evolution of two sexes. Proc. R. Soc. B 279, 1865–1872 10.1098/rspb.2011.1871 (doi:10.1098/rspb.2011.1871) PMC3297446 22158961

[104] Lane N 2011 Mitonuclear match: Optimizing fitness and fertility over generations drives ageing within generations. Bioessays 33, 860–869 10.1002/bies.201100051 (doi:10.1002/bies.201100051) 21922504

[105] Zouros E Ball AO Saavedra C Freeman KR 1994 An unusual type of mitochondrial-DNA inheritance in the blue mussel mytilus. Proc. Natl Acad. Sci. USA 91, 7463–7467 10.1073/pnas.91.16.7463 (doi:10.1073/pnas.91.16.7463) 8052604 PMC44421

[106] Howard CV Reed MG 2010 Unbiased stereology: three-dimensional measurement in microscopy, 2nd edn Liverpool: QTP Publications

